# Supplementary material for: The cost-effectiveness of common strategies for the prevention of transmission of SARS-CoV-2 in universities
Source: PLoS One. 2021 Sep 30;16(9):e0257806. doi: 10.1371/journal.pone.0257806 (PMC8483333; doi:10.1371/journal.pone.0257806)
Supplement: S1 Appendix — (DOCX) [file pone.0257806.s001.docx]

# Online Appendix

**S1 Table. Student preferences regarding re-opening universities in the Fall of 2020.**

**S1 File. Additional information on interventions and use of the online model.**

**S2 File. Model equations for estimating the probability of super-spreader event.**

**S2 Table.** **Lists of all parameters including total costs and probabilities used as model inputs for estimating the cost-effectiveness of strategies to improve infection control for Covid-19 in a university setting with 16,000 students and 4,500 employees on campus during a 90-day semester.**

**S3 Table.** **Cost-effectiveness outcomes in terms of incremental costs per infection averted for strategies to improve infection control for Covid-19 in universities at the prevalence of actively infectious cases of 0.131% in the community.**

**S1 Figure. The cost-effectiveness plane, representing incremental costs vs. incremental QALYs, for multiple interventions of our model compared to implementing CDC guidelines alone (depicted at prevalence of 0.1% of infectious cases).**

**S2 Figure. Cost-effectiveness acceptability curves. The figure shows the probability of cost-effectiveness for each intervention at different willingness-to-pay values at a prevalence of actively infectious cases of 0.1%.**

**S3 Figure. Multi-way sensitivity analysis identifying the most cost-effective intervention at different values of the number of close contacts between students on campus, transmission rate per close student contact, and willingness-to-pay at a 1% prevalence of actively infectious cases.**

**S4 Figure. Multi-way sensitivity analysis identifying the most cost-effective intervention at different values of the number of close contacts between students on campus, transmission rate per close student contact, and willingness-to-pay at a 2% prevalence of actively infectious cases.**

**S5 Figure. Multi-way sensitivity analysis identifying the most cost-effective intervention at different values of community compliance with wearing face mask, prevalence of actively infectious cases, and willingness-to-pay threshold.**

**S1 Table.** Student preferences regarding re-opening universities in the Fall of 2020. Graduate students who had both attended in-person classes during the semester and online classes during the lockdown in the Spring of 2020 were identified by departmental administrators in Health Policy and Management in a convenience sample. A total of 46 students participated. Details of the exercises can be found [here](https://www.medrxiv.org/content/10.1101/2020.08.26.20182352v1).

| **Average risk of infection accepted until preference of online class** | **Students willing to attend social gatherings given low/moderate prevalence of COVID-19 in NYC** | **Average number of times per week going off-campus** | **Average annual tuition willing to pay for online classes** |
| --- | --- | --- | --- |
| **23%** | **28%** | **$9,818** | **3.6** |

| **Risk of infection accepted until preference of online class** | **Willingness to attend social gatherings** | **Number of times per week going off campus** | **Annual tuition willing to pay for online classes** |
| --- | --- | --- | --- |
| 0.125 | No | 0.5 | 9050 |
| 0.125 | Low | 5 | 10000 |
| 0.03 | No | 4 | 7000 |
| 0.003 | No | 2 | 10000 |
| 0.475 | Low | 6 | 10000 |
| 0.175 | Medium | 10 | 10000 |
| 0.0075 | No | 1 | 16000 |
| 0.275 | No | 2 | 5000 |
| 0.03 | No | 1 | 15000 |
| 0 | No | 10 | 17000 |
| 0.175 | No | 5 | 10000 |
| 0.175 | High | 3 | 10000 |
| 0.075 | High | 8 | 7500 |
| 0.225 | No | 2 | 15000 |
| 0.03 | Medium | 3 | 17000 |
| 0.003 | No | 1 | 5000 |
| 0.075 | No | 2 | 12000 |
| 0.375 | No | 2 | 8000 |
| 0.775 | Medium | 5 | 10000 |
| 0.175 | Medium | 10 | 10000 |
| 0.575 | No | 4 | NA |
| 1 | No | 1 | 0 |
| 0.125 | No | 3 | 10000 |
| 0.075 | No | 0 | 15000 |
| 0.375 | Low | 1 | 10000 |
| 0.003 | No | 0 | 5000 |
| 0.125 | Low | 3 | 5000 |
| 0.175 | Medium | 2 | 10000 |
| 0.03 | Low | 2 | 10000 |
| 0.725 | No | 0 | 5000 |
| 1 | High | 4 | 10000 |
| 0.125 | Medium | 3 | 10000 |
| 0.0075 | No | 1 | 8000 |
| 0.175 | Low | 4 | 10000 |
| 0.003 | No | 0 | 10000 |
| 0.875 | High | 3 | 10000 |
| 0.03 | No | 1 | 10000 |
| 0.225 | No | 3 | 10000 |
| 0.0075 | High | 2 | 10200 |
| 0.03 | No | 2 | 15050 |
| 0.275 | High | 2 | 15000 |
| 0.225 | High | 7 | 15000 |
| 0.003 | No | 5 | 5000 |
| 1 | No | 21 | 10000 |
| 0.03 | No | NA | 5000 |
| 0.075 | No | 4 | 5000 |

**S1 File. Additional information on interventions and use of the online model.**

*Estimating the prevalence of actively infectious cases in the community*

Users who wish to model outcomes for their local university should use predicted rates of prevalent infectious cases /100,000 population in the community surrounding their university from local models at the time of opening and enter these into the online model.[1] Users should be careful to ensure that they enter case numbers that are adjusted for under-reporting and for the duration of infectious illness. Cornell and Harvard University have developed predictive analytics that can spot changes in the R_0_ for a given area.[2] Alternatively, it is possible to obtain estimates of the active infectious prevalence from your local health department.

Users should also be careful to change assumptions surrounding the number of contacts between students, students and staff/faculty, between faculty/staff, and between faculty/staff or students with the surrounding community. Ideally, this would be obtained from student survey data. Areas that have a lower level of concern regarding the dangers of COVID-19 may have more close contacts with others and lower rates of mask use. It is therefore important to adjust for both the number of close contacts and the local prevalence of mask use.[3]

**CDC guidelines**

The CDC [provides guidance](https://www.cdc.gov/coronavirus/2019-ncov/community/colleges-universities/index.html) on procedures that should be put into place prior to re-opening a university.[4] These include mask use, social distancing, improved ventilation, and cleaning measures. At the time of our analysis, the CDC recommended against testing, citing a lack of sufficient evidence. The CDC updated its guidance in early 2021 to read, “CDC offers considerations for ways in which IHEs can help protect students, faculty, and staff and slow the spread of COVID-19. Testing to diagnose COVID-19 is one component of a comprehensive strategy and should be used in conjunction with promoting behaviors that reduce spread, maintaining healthy environments, maintaining healthy operations, and preparing for when someone gets sick.”[5]^(3rd paragraph)^

**Enhanced Mask Use**

In July of 2020, roughly 80% of Americans self-reported social distancing and wearing masks most of the time when indoors, and 59% reported wearing masks consistently.[3] These results were obtained by a large but non-representative sample of the United States. Local use can be found on an [interactive map](https://www.nytimes.com/interactive/2020/07/17/upshot/coronavirus-face-mask-map.html) and can be entered into the [R-shiny interface](https://openupuniversities.shinyapps.io/shinyapp_server/).

Cotton masks are widely in use, and may reduce transmission by 67% (0.33 adjusted odds ratio).[6, 7] The quality of masks in use by students may be lower, and the fit may not be a snug. For this reason, universities may wish to supply students with masks. The online model allows the user to define the mask cost and efficacy.

N95 masks are expensive and uncomfortable. However, they are also highly effective, reducing infection by over 90%.[6] The masks supplied by Columbia University are 2-ply and are estimated to be roughly 80% effective at preventing infection with COVID-19 (relative to 67% for masks in prevalent use and 94% for N95 masks).[6]

Not all affiliates appear to use the provided masks, and the university must nevertheless purchase disposable “back up” masks for use by affiliates who forget to bring a mask upon coming to campus. Therefore, we assume that providing these masks will come at their bulk price cost ($2/mask at Columbia University) and that this cost comes over and above what the university would otherwise have spent on masks.

**Temperature Screening Cameras**

We examined the cost-effectiveness of highly sensitive thermal imaging cameras to be place in the 10 highest foot traffic areas on campus. We assumed 20 guards would operate the cameras, each working 8 hours per day on weekdays. On weekends the cameras would be operated by existing security staff. We used [FLIR Systems A700](https://www.flir.com/landing/instruments/The-Complete-Guidebook-on-Thermal-Screening?creative=449344198072&keyword=flir%20camera%20for%20fever&matchtype=e&network=g&device=c&gclid=Cj0KCQjwg8n5BRCdARIsALxKb962tZZdQln-c4h0TYP8QR51nKzghpdcEaCycdrxj6U0sQ5HTiwmF3oaAhz9EALw_wcB) as a unit as it is standard in the industry (FLIR Systems, Sweden) programmed for temperature screening.

Because these cameras screen every affiliate on campus for fever every day, they tend to remove affiliates from the campus irrespective of whether they are infected with COVID-19. The results in *de facto* social distancing, and therefore proves to be an effective mechanism for reducing the spread of COVID-19 even if no cases are present. It also reduces on-campus exposure to influenza-like illnesses, thus carrying additional benefits.

**Infection Hospitalization Rate and Infection Fatality Rate**

The infection fatality rate (IFR) and infection hospitalization rate (IHR) can vary greatly by locality.[8, 9] We obtained age-specific IFRs from the literature for the US as a whole,[9] and then computed a weighted average rate using the age distribution of students and faculty separately at Columbia University.

For IHRs, we used CDC data to apportion hospitalization risk by age.[10] We then used the age distribution of affiliates at Columbia University to compute a weighted average IHR using a mean US rate from the literature.[9]

While other universities will have different age and risk distributions, we find that including or excluding those over the age of 70 at Columbia University had little impact on our weighted mean values.

**S2 File. Model equations for estimating the probability of super-spreader event.**

*Model equations*

For each susceptible student and staff/faculty, the probability of becoming infected outside of campus was calculated as follows:

$P\left[ infection outside of campus | simulation unit i \right]=\left( 1-\left( 1-p_{c}.r \right)^{c_{o}^{i}} \right),$

where $p_{c}$ represents the prevalence of infectious COVID-19 cases in the local community outside of campus; $r$ is the transmission rate per close contact; and $c_{o}^{i}$ represents the average number of daily close contacts that each simulation unit $i$ (students or staff/faculty) makes in the local community outside of campus.

To account for the proportion of population wearing face masks outside of campus, we assigned a multiplier factor,$1-C_{o}.RR_{wearing mask}$; where $C_{o}$ represents the compliance rate with wearing face masks in the NYC-defined neighborhoods within which the university resides; and $RR_{wearing mask}$represents the risk reduction associated with wearing face masks.

Similarly, the probability of becoming infected inside the campus was calculated as follows:

${P\left[ infection inside campus | simulation unit i \right]=1-\left( 1-p_{s}\left( t \right).r \right)}^{c_{s}^{i}}.\left( 1-p_{e}\left( t \right).r \right)^{c_{e}^{i}}$;

where $p_{s}\left( t \right)$ and $p_{e}\left( t \right)$ represents the prevalence of infectious cases among students, and staff/faculty, respectively, at time $t$; $r$ is the transmission rate per close contact; and $c_{s}^{i}$, and $c_{e}^{i}$ represents the average number of close contacts that each simulation unit $i$ makes with students and staff/faculty, on campus, respectively.

In addition, we modeled the probability of a super-spreader event based upon the prevalence of infectious cases of disease in the community, $p_{c}$, a standard gamble-based risk assessment administered to students that revealed students’ preferences for participation in community parties, $p_{party}$ as the daily probability of students’ participation in a community party, and the average number of attendees in a community party, $N_{party}$, as follows:

$$P\left[ super spreader event \right]=p_{party}.\left( 1-\left( 1-0.1*p_{c}\left( t \right) \right)^{N_{party}} \right).$$

Because roughly 10% of exposed cases are, on average, responsible for 80% of subsequent infections,[11] we multiplied $p_{c}$ by 0.1 to approximate the prevalence of super-spreaders in a community party. Therefore, we modeled the probability of having at least one super-spreader out of $N_{party}$ participants in the party.

**S2 Table.** Lists of all parameters including total costs and probabilities used as model inputs for estimating the cost-effectiveness of strategies to improve infection control for Covid-19 in a university setting with 16,000 students and 4,500 employees on campus during a 90-day semester.

| **Parameters** | **Baseline** | **Distribution*** | **Source** |
| --- | --- | --- | --- |
| *Population* |  |  |  |
| Number of students on campus | 16,000 | - | Data from Columbia University |
| Number of staff/faculty on campus | 4,500 | - | Data from Columbia University |
| *Daily number of close contacts* |  |  |  |
| Between each student and other students on campus (but not in dorms) | 10 | Gamma (25, 2.5) | Expert opinion† |
| Between each student and staff/faculty on campus | 1 | Gamma (4, 4) | Expert opinion† |
| Between each student and community members outside of campus | 2 | Gamma (4, 2) | Expert opinion† |
| Between each staff/faculty and students on campus | 4 | Gamma (16, 4) | Expert opinion† |
| Between each staff/faculty and other staff/faculty on campus | 1 | Gamma (4, 4) | Expert opinion† |
| Between each staff/faculty and community members outside of campus | 2 | Gamma (16, 8) | Expert opinion† |
| *Probabilities and rates* |  |  |  |
| Transmission rate per close contact | 0.066 | Normal (0.066, 0.005) | [12] |
| Proportion of affiliates immune to COVID-19 | 0.06 | Triangular (0, 0.2, 0.06) | [13] |
| Progression time for Covid-19 |  |  |  |
| Incubation time ($r_{inc}$) | 5 days | Triangular (3, 14, 5) | [14, 15] |
| Time from infectiousness to symptoms onset ($r_{s}$) | 2 days | Triangular (1, 3, 2) | [14, 15] |
| Time from exposure to infectiousness | 3 days | Probability distribution of $r_{inc}$ - probability distribution of $r_{s}$ | [14, 15] |
| Duration of infectiousness after symptoms onset | 10 days | Triangular (6, 14, 10) | [14-17] |
| Proportion of asymptomatic cases among all exposed people (excluding the ones initially asymptomatic but became symptomatic eventually) | 0.47 | Beta (52.53, 59.23596) | [18] |
| Infection hospitalization rate among students | 0.008 | Beta (99.192, 12299.81) | [9, 10] |
| Infection hospitalization rate among staff/faculty | 0.018 | Beta (98.182, 5356.374) | [9, 10] |
| Infection fatality rate among students | 0.0002 | Beta (99.9798, 499799) | [9] |
| Infection fatality rate among staff/faculty | 0.0015 | Beta (99.8485, 66465.82) | [9] |
| Probability of long COVID-19[19] | 0.133 | Beta (86.567, 564.313) |  |
| Proportion of students’ compliance with stay-home order when they notice their symptoms | 0.85 | Triangular (0.75, 0.9, 0.85) | Assumption |
| Proportion of community members’ compliance with wearing masks outside of campus | 0.78 | Triangular (0.72, 0.78, 0.78) | [3, 20] |
| *Direct costs (U.S. dollars in 2020 USD)* |  |  |  |
| Hospitalization | $23,489 | - | [21, 22] |
| Funeral cost for infection death | $10,000 | Gamma (16, 0.0016) | Assumption |
| Intervention costs¶ |  |  |  |
| CDC guidelines |  |  | [4] |
| Adhering to cleaning protocol costs | $318,798 | - | [23] |
| Custodial staff | $979,503 | - | [23] |
| Personal protective equipment | $1,386,898 | - | [23] |
| Enhanced masks | $164,000 | - | 2 masks/affiliate @$2mask. Enhanced masks are provided on top of regular masks. The number here only reflects the cost of providing enhanced masks. |
| Temperature cameras | $485,000 | - | 10 units @ $12,000/unit and installment cost of $500/unit. In addition, assuming wage for 20 staff operating 8 hrs/day over the entire semester at the rate of $25/hr. ¶ |
| PCR test (per test) | $45 | - | Internal data for test cost. In addition, assumed personnel time value for 4 personnel at $25/hr for 8 hrs/day for 1500 tests; 30% administrative costs; 10 cents shipping/specimen. ¶ |
| *Indirect costs (U.S. dollars in 2020 USD)* |  |  |  |
| Productivity loss for COVID-19 infection without hospitalization for symptomatic employee who either got detected or self-quarantined (per person over 2 weeks) | $2,800 | Gamma (100, 0.03571429) | Assuming 14 days of quarantine at $25/hr for 8 hrs/day |
| Productivity loss for infection hospitalization among employees (per person over 3 weeks) | $4,200 | Gamma (100, 0.02380952) | Assuming 21 days of quarantine at $25/hr for 8 hrs/day |
| Productivity loss for infection death per student (this value adjusts for the average student age at death and lifetime losses of average salary of $50,000/year until the age of 65; future values are discounted at 3%) | $ 1,190,411 | Gamma (100, 8.40046e-05) | Assuming 39 yrs (65-26) work years lost at $50,000/yr. 26 represents the average age of students at Columbia University |
| Productivity loss for infection death per staff/faculty (this value adjusts for the average staff/faculty age at death and lifetime losses of average salary of $50,000/year until the age of 65; future values are discounted at 3%) | $766,190 | Gamma (100, 0.0001305159) | Assuming 19 yrs (65-46) work years lost at $50,000/yr. 46 represents the average age of staff/faculty at Columbia University |
| Lost tuition per day for online vs. in -person classes (per student) | $46 | - | Calculated from a student survey average tuition for the Fall of 2020 semester at Columbia University |
| Productivity loss associated with a false-positive test result (per staff/faculty per day) | $450 | Gamma (100, 0.2222222) | Assuming losing 8 hours of work productivity and 10 hours of leisure time at $25/hr |
| *Intervention effects* |  |  |  |
| CDC guidelines |  |  |  |
| Incidence rate ratio of infection for frequent hand washing/sanitizing | 0.64 | - | [24, 25] |
| Odds ratio of infection for regular mask use | 0.33 | - | [6, 24] |
| Overall effect (odds ratio) | 0.21 | Beta (78.669, 293.816) | [6, 24, 25] |
| Symptom checking application |  |  |  |
| Percentage points change in proportion of students’ compliance with stay-home order when they notice their symptoms | 10% | Triangular (0.75, 0.9, 0.85)+0.1 | Assumption |
| Standardized masks |  |  |  |
| Odds ratio of infection for standardized masks | 0.2 | - | Assuming average effects of regular masks and N95 masks [6, 24] |
| Overall effects of standardized masks and frequent hand washing/sanitizing (odds ratio of infection) | 0.128 | Beta (87.072, 593.178) | [6, 24] |
| Temperature camera (fever) | In the model, even without any interventions, 85% of symptomatic affiliates were assumed to quarantine upon noticing their symptoms. The additional effect of thermal cameras was modeled assuming that the other 15% of symptomatic affiliates would at one point over the course of their infectiousness would be detected by thermal cameras.  For the specificity, we calculated the daily incidence of flu by dividing the total number flu cases in the US in 2018-2019 by the population size and number of days during a 26-week flu season as follows:  35520883/(329.45*10^6)/(26*7)~0.001; where 35520883 was the number flu cases in the US in 2018-2019.[26] Given the 0.001 daily incidence of flu, we assumed a false positive rate of 0.001 (specificity of 0.999). To reduce the number of false positive screens, we assumed those who screened positive by thermal cameras were subsequently checked with a tympanic membrane thermometer. | | |
| Test for SARS-CoV-2 |  |  |  |
| Sensitivity | 0.95 | - | [27] |
| Specificity | 1 | - | [27] |
| *Health-related quality of life* |  |  |  |
| Losses of QALYs associated with a COVID-19 symptomatic case | 0.008 | Beta (99.192, 12299.81) | [28] |
| Losses of QALYs associated with a long COVID-19 infection | 0.034 | Beta (96.566, 2743.61) | [28] |
| Losses of QALYs associated with a COVID-19 hospitalization | 0.020 | Beta (97.970, 4776.154) | [28] |
| Losses of QALYs associated with a COVID-19 death among student population (adjusted for average age at death, age-dependent QALYs of the US general population, and discounting future values at 3%) | 23.94 | Normal (23.94, 2.40) | Assuming an average age student (26 years) would have otherwise lived for 80 years at age-dependent QALYs of the general US population.[29] |
| Losses of QALYs associated with a COVID-19 death among employee population (adjusted for average age at death, age-dependent QALYs of the US general population, and discounting future values at 3%) | 18.33 | Normal (18.33, 1.83) | Assuming an average age staff/faculty (46 years) would have otherwise lived for 80 years at age-dependent QALYs of the general US population[29] |

Note: A close contact is defined as person-to-person contact < 6 feet for > 10 minutes.

* For triangular distributions, the parameters listed are lower limit, upper limit, and mode; for normal distributions, parameters are mean and standard deviation; for beta distribution, parameters are shape 1 and shape 2; and for gamma distributions, parameters are shape and rate.

†Expert opinion based on video conferences with the Public Health Committee at Columbia University, which is comprised of a range of infectious disease experts and administrators.

¶Costs reflect actual costs paid by Columbia University including personnel.

**S3 Table.** Cost-effectiveness outcomes in terms of incremental costs per infection averted for strategies to improve infection control for Covid-19 in universities at the prevalence of actively infectious cases of 0.131% in the community.

| **Interventions** | **Days open** | **Costs ($)** | **Incremental costs ($)** | **Infections** | **Infections averted** | **ICER ($/infection averted)** |
| --- | --- | --- | --- | --- | --- | --- |
| CDC guidelines | 79 (37, 90) | 11557117 (2799287, 42347542) | Reference | 482 (62, 1054) | Reference | Reference |
| Symptom-checking mobile application plus CDC guidelines | 81 (40, 90) | 9655276 (2798787, 40553897) | -1901841 (-8325490, 1835) | 437 (58, 1026) | 45 (5, 122) | -41874 (-105170, 315) |
| Thermal cameras plus CDC guidelines | 81 (41, 90) | 13001005 (6401482, 42457308) | 3345729 (824777, 4622951) | 430 (56, 1016) | 6 (-15, 30) | 527674 (-7120743, 8073031) |
| Gateway testing plus CDC guidelines | 83 (47, 90) | 8957984 (3707797, 36104540) | -4043021 (-11416977, -1863169) | 388 (21, 950) | 43 (-12, 185) | -94900 (-1485491, 1693107) |
| Standardized masks plus CDC guidelines | 89 (72, 90) | 3803800 (2845555, 16328341) | -5154184 (-26890309, -851424) | 236 (31, 696) | 151 (-18, 466) | -34085 (-95280, 117971) |
| Weekly testing plus CDC guidelines | 90 (90, 90) | 14039473 (13861122, 14249998) | 10235673 (-2162557, 11062938) | 152 (17, 373) | 85 (10, 375) | 120722 (-5320, 1083835) |
| Package intervention | 90 (90, 90) | 14080432 (13941823, 14272649) | 40958 (2811, 82009) | 129 (16, 309) | 23 (0, 71) | 1809 (42, 263049) |

Note: Negative ICERs in this table represent a cost-saving scenario, indicating the comparator saves money and improves health compared with the baseline intervention.

**S1 Figure**. The cost-effectiveness plane, representing incremental costs vs. incremental QALYs, for multiple interventions of our model compared to implementing CDC guidelines alone (depicted at prevalence of 0.1% of infectious cases).





**S2 Figure**. Cost-effectiveness acceptability curves. The figure shows the probability of cost-effectiveness for each intervention at different willingness-to-pay values at a prevalence of actively infectious cases of 0.1%.


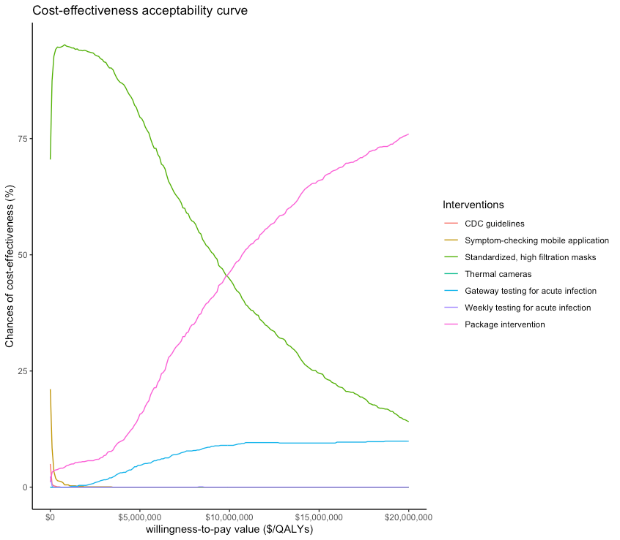


**S3 Figure**. Multi-way sensitivity analysis identifying the most cost-effective intervention at different values of the number of close contacts between students on campus, transmission rate per close student contact, and willingness-to-pay at a 1% prevalence of actively infectious cases.


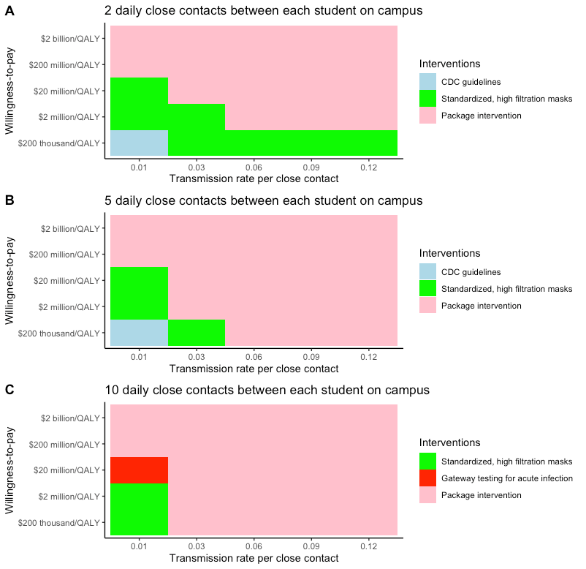


**S4 Figure**. Multi-way sensitivity analysis identifying the most cost-effective intervention at different values of the number of close contacts between students on campus, transmission rate per close student contact, and willingness-to-pay at a 2% prevalence of actively infectious cases.


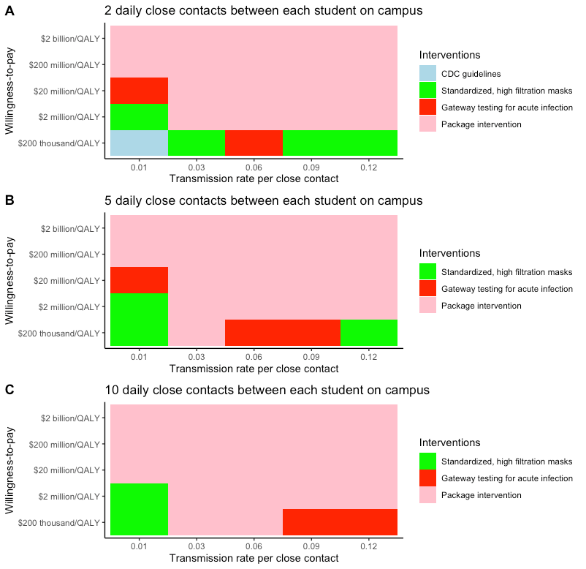


**S5 Figure**. Multi-way sensitivity analysis identifying the most cost-effective intervention at different values of community compliance with wearing face mask, prevalence of actively infectious cases, and willingness-to-pay threshold.


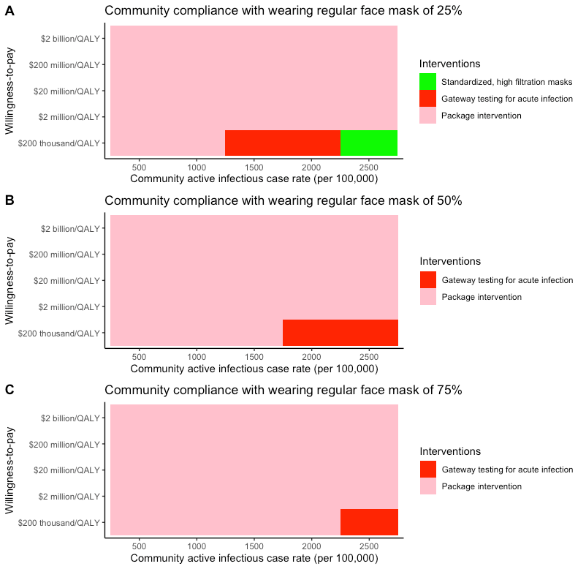


**References**

1. Muennig P, Zafari Z. OpenUp Model. Mailman School of Public Health. Columbia University. Available online at: <https://www.publichealth.columbia.edu/academics/departments/health-policy-and-management/openup-model> Accessed 8/2/2020. 2020.

2. Liu D, Clemente L, Poirier C, Ding X, Chinazzi M, Davis JT, et al. A machine learning methodology for real-time forecasting of the 2019-2020 COVID-19 outbreak using Internet searches, news alerts, and estimates from mechanistic models. arXiv preprint arXiv:200404019. 2020.

3. Katz J, Katz MS, Quealy K. A detailed map of who is wearing masks in the U.S. Available online at: <https://www.nytimes.com/interactive/2020/07/17/upshot/coronavirus-face-mask-map.html> Accessed 7/28/2020. 2020.

4. Centers for Disease Control and Prevention. Reopening Guidance for Cleaning and Disinfecting Public Spaces, Workplaces, Businesses, Schools, and Homes. Available online at: <https://www.cdc.gov/coronavirus/2019-ncov/community/colleges-universities/considerations.html> Accessed 1/4/2021.

5. Centers for Disease Control and Prevention. Testing, screening, and outbreak response in institutions of higher educaton (IHE). Available online at: <https://www.cdc.gov/coronavirus/2019-ncov/community/colleges-universities/ihe-testing.html> Accessed 1/22/2021.

6. Chu DK, Akl EA, Duda S, Solo K, Yaacoub S, Schünemann HJ, et al. Physical distancing, face masks, and eye protection to prevent person-to-person transmission of SARS-CoV-2 and COVID-19: a systematic review and meta-analysis. The Lancet. 2020.

7. Lustig SR, Biswakarma JJH, Rana D, Tilford SH, Hu W, Su M, et al. Effectiveness of Common Fabrics to Block Aqueous Aerosols of Virus-like Nanoparticles. ACS nano. 2020;14(6):7651-8. Epub 2020/05/29. doi: 10.1021/acsnano.0c03972. PubMed PMID: 32438799.

8. Yang W, Kandula S, Huynh M, Greene SK, Van Wye G, Li W, et al. Estimating the infection fatality risk of COVID-19 in New York City, March 1-May 16, 2020. medRxiv. 2020:2020.06.27.20141689. doi: 10.1101/2020.06.27.20141689.

9. Chen X, Hazra DK. Understanding the Bias between the Number of Confirmed Cases and Actual Number of Infections in the COVID-19 Pandemic. medRxiv. 2020.

10. Centers for Disease Control and Prevention. COVID-NET. COVID-19 Laboratory-Confirmed Hospitalizations. Available online at: <https://gis.cdc.gov/grasp/COVIDNet/COVID19_5.html>. Accessed 8/6/2020.

11. Endo A, Abbott S, Kucharski AJ, Funk S. Estimating the overdispersion in COVID-19 transmission using outbreak sizes outside China. Wellcome Open Research. 2020;5(67):67.

12. Bi Q, Wu Y, Mei S, Ye C, Zou X, Zhang Z, et al. Epidemiology and transmission of COVID-19 in 391 cases and 1286 of their close contacts in Shenzhen, China: a retrospective cohort study. The Lancet Infectious Diseases. 2020.

13. Cneters for Disease Control and Prevention. Large-scale Geographic Seroprevalence Surveys. Availale online at: <https://www.cdc.gov/coronavirus/2019-ncov/cases-updates/geographic-seroprevalence-surveys.html> Accessed 8/05/2020.

14. He X, Lau E, Wu P, Deng X, Wang J, Hao X, et al. April 2020, posting date. Temporal dynamics in viral shedding and transmissibility of COVID-19. Nat Med doi. 10.

15. World Health Organization. Available online at: <https://www.who.int/docs/default-source/coronaviruse/situation-reports/20200402-sitrep-73-covid-19.pdf?sfvrsn=5ae25bc7_2#:~:text=The%20incubation%20period%20for%20COVID,occur%20before%20symptom%20onset> Accessed 8/5/2020.

16. February A. Interim Guidance on Duration of Isolation and Precautions for Adults with COVID-19.

17. Walsh KA, Spillane S, Comber L, Cardwell K, Harrington P, Connell J, et al. The duration of infectiousness of individuals infected with SARS-CoV-2. J Infect. 2020.

18. Sakurai A, Sasaki T, Kato S, Hayashi M, Tsuzuki S-i, Ishihara T, et al. Natural History of Asymptomatic SARS-CoV-2 Infection. N Engl J Med. 2020. doi: 10.1056/NEJMc2013020.

19. Sudre CH, Murray B, Varsavsky T, Graham MS, Penfold RS, Bowyer RC, et al. Attributes and predictors of long COVID. Nat Med. 2021 Apr;27(4):626–31. .

20. Arp NL, Nguyen TH, Graham Linck EJ, Feeney AK, Schrope JH, Ruedinger KL, et al. Use of face coverings by the public during the COVID-19 pandemic: an observational study. medRxiv. 2020:2020.06.09.20126946. doi: 10.1101/2020.06.09.20126946.

21. Avalere. COVID-19 Hospitalizations Projected to Cost up to $17B in US in 2020. Available online at: <https://avalere.com/insights/covid-19-hospitalizations-projected-to-cost-up-to-17b-in-us-in-2020>. Accessed 8/6/2020.

22. Bartsch SM, Ferguson MC, McKinnell JA, O’Shea KJ, Wedlock PT, Siegmund SS, et al. The Potential Health Care Costs And Resource Use Associated With COVID-19 In The United States: A simulation estimate of the direct medical costs and health care resource use associated with COVID-19 infections in the United States. Health Aff (Millwood). 2020:10.1377/hlthaff. 2020.00426.

23. Association of School Business Associates International. What will it cost to re-open schools? Available online at: <https://www.asbointl.org/asbo/media/documents/Resources/covid/COVID-19-Costs-to-Reopen-Schools.pdf> Accessed 8/3/2020.

24. Jefferson T, Foxlee R, Del Mar C, Dooley L, Ferroni E, Hewak B, et al. Physical interventions to interrupt or reduce the spread of respiratory viruses: systematic review. BMJ. 2008;336(7635):77-80.

25. Beale S, Johnson AM, Zambon M, Hayward AC, Fragaszy EB. Hand Hygiene Practices and the Risk of Human Coronavirus Infections in a UK Community Cohort. Wellcome Open Research. 2020;5(98):98.

26. Centers for Diseasse Control and Prevetnion. Estimated influenza illnesses, hospitalizations, and deaths in the United States. 2018-2019 Infuenza season. Available online at: <https://www.cdc.gov/flu/about/burden/2018-2019.html> Accessed 1/28/2020.

27. Broad Institute. Fall college testing program. Available online at: <https://covid-19-test-info.broadinstitute.org/safe-for-school/>. Accessed 8/6/2020.

28. Sandmann FG, Davies NG, Vassall A, Edmunds WJ, Jit M, Sun FY, et al. The potential health and economic value of SARS-CoV-2 vaccination alongside physical distancing in the UK: a transmission model-based future scenario analysis and economic evaluation. The Lancet Infectious Diseases. 2021.

29. Briggs A. Moving beyond “lives saved” from COVID-19. Available online at: <https://avalonecon.com/moving-beyond-lives-saved-from-covid-19/>. Accessed 8/5/2020.
