# Supplementary material for: The cost-effectiveness of common strategies for the prevention of transmission of SARS-CoV-2 in universities
Source: PLoS One. 2021 Sep 30;16(9):e0257806. doi: 10.1371/journal.pone.0257806 (PMC8483333; doi:10.1371/journal.pone.0257806)
Supplement: Attachment — Submitted filename: Point-by-point Response.docx [file pone.0257806.s004.docx]

Dear Dr. Schwartzman,

Thank you very much for the opportunity to respond to the minor comments. We have responded to each comment in a point-by-point response letter below.

Thank you for your time and help with our manuscript.

Best,

Peter Muennig and Zafar Zafari

Reviewer #1: Thanks to the authors for addressing my comments. Only a few minor comments remain:

**[Response] We would like to thank the reviewer for his/her excellent comments and help throughout the revisions process. Your comments throughout this process have greatly improved our paper. Thank you again for your time and invaluable comments/feedback.**

-Repeat word of "iterations" in the first sentence of Analyses

**[Response] Thank you. We have corrected this problem, which appears to be an error in PDF conversion.**

-Repeat word "actively" in the third sentence of Analyses

**[Response] Thank you. As above, this appeared to be a conversion issue.**

-In Table 3 - how should readers interpret negative ICERs? I suggest a footnote to explain this or to remove confidence intervals around ICERs since confidence intervals require context for interpretation (e.g., you can have a negative ICER in quadrant II and IV, and a positive ICER in quadrant I and III -- though interpretation of positive and negative ICERs vary GREATLY depending on quadrant). In the supplement, it appears most results fall in quadrant I and II. Please provide more clarity on interpretation in a footnote or text.

**[Response] Thank you. As suggested, we removed the intervals around the ICERs in Table 3 and added a footnote that describes the meaning of negative ICERs in this table.**
